# Assessment of ultrasound-assisted vacuum impregnation as a method for modifying cranberries’ quality

**DOI:** 10.1016/j.ultsonch.2022.106117

**Published:** 2022-08-08

**Authors:** Dominik Mierzwa, Justyna Szadzińska, Bartosz Gapiński, Elżbieta Radziejewska-Kubzdela, Róża Biegańska-Marecik

**Affiliations:** a Division of Process Engineering, Institute of Chemical Technology and Engineering, Poznań University of Technology, ul. Berdychowo 4, 60-965 Poznań, Poland; b Division of Metrology and Measurement Systems, Institute of Mechanical Technology, Poznań University of Technology, ul. Jana Pawła II 24, 60-965 Poznań, Poland; c Department of Food Technology of Plant Origin, Poznan University of Life Sciences, ul. Wojska Polskiego 31, 60-624 Poznań, Poland

**Keywords:** Berry fruit, Fortified food, Ascorbic acid, X-ray microtomography, Color, Antioxidants

## Abstract

Combined vacuum impregnation and ultrasound was proposed as an alternative method to improve the infusion of ascorbic acid in berry fruit. The effect of ultrasound application at different stages of impregnation – vacuum, relaxation, and both stages – on the qualitative characteristics of impregnated cranberries was investigated. The quality assessment was based on porosity, color, antioxidant capacity, anthocyanin, polyphenol and structure compound content. Ultrasound-assisted vacuum impregnation contributed to higher ascorbic acid content, smaller relative color difference, and greater antioxidant properties. It was found that the degree of impregnation varies considerably and depends on the stage of using ultrasound. Due to more favorable quality attributes, the conclusion was reached that ultrasound should be applied during the relaxation stage of vacuum impregnation.

## Highlights

• The whole cranberry fruit was impregnated.
• Ultrasound intensified the mass transfer during vacuum impregnation.
• The degree of impregnation depends on the stage in which ultrasound was used.
• No negative impact of vacuum impregnation on quality was observed.

## Graphical abstract

**Figure:**
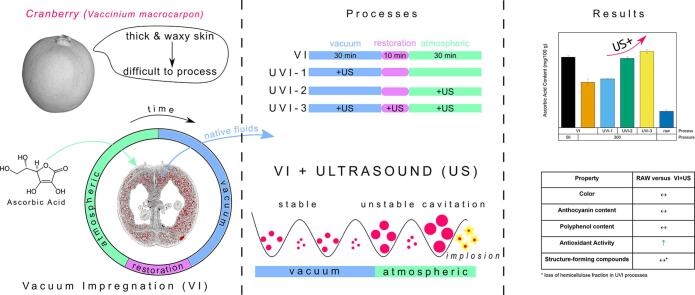

## 1 Introduction

One potential and also promising method for fortifying food with vitamins, minerals, and other biologically active substances is vacuum impregnation (VI). In this process, the native fluids are removed from the biological tissue under reduced pressure, and then replaced with a specific solution, an *impregnant* (e.g., isotonic or hypertonic), which contains a functional ingredient. The application of an isotonic solution allows for the effective introduction of components into the tissue without its deformation (shrink or shrivel) as appears in a hypertonic solution or swell in a hypotonic solution. Furthermore, the reduction of the content of soluble solids in the tissue of raw material impregnated in hypotonic solution can result in unfavorable changes in sensory quality [1].

The mechanism for mass transfer of the solution into the capillary-porous structure of the food product is based mainly on the pressure gradient, and consists of the two following steps: vacuum and relaxation [2]. Chiralt and Fito [3] concluded that the primary phenomenon in vacuum impregnation was the hydrodynamic mechanism (HDM), due to capillary action and pressure gradients as a consequence of internal volume changes, i.e., the deformation-relaxation phenomena (DRP). However, the intensity of these phenomena is strongly dependent on the vacuum pressure, relaxation time, food microstructure, and mechanical properties of the biological matrix [3], [4]. Due to the benefits that follow from vacuum impregnation, such as quality improvement and an increased mass transfer, this method has found wide application in food processing, for example, in osmotic dehydration, shelf-life prolongation, functional food production, and also as a pre-treatment for various processes, such as, freezing, canning, and drying [1], [5], [6].

Fito et al. [7] reported that the efficiency of vacuum impregnation depends to a significant degree on tissue structure and pore characteristics, which are unique to each food product. The smaller the porosity or pore diameter, the less effective the impregnation, and conversely, the higher the porosity the higher the level of impregnation [7]. Knowing the porosity of a given food product allows the amount of impregnant that can be introduced into the pores to be estimated. Fruit and vegetables are characterized by relatively high porosity compared to fish and other food products; thus, they are particularly attractive in relation to the impregnation process [8].

If the fruit is processed while whole, an important element that significantly affects the mass transfer is its skin. In some fruit, like cranberries, the outer surface consists of thick, dense epicarp that is coated with cutin and natural waxes. These substances are characterized by their insolubility or poor water solubility, which is reflected in very low permeability. The skin plays a major role in the control of transpiration, and protects the fruit against weather conditions (e.g., dehydration), insects, and parasites. However, the waxy cuticle hinders mass transfer and makes processing (e.g., osmotic dehydration, drying, or impregnation) very difficult [9]. Thick-skinned fruits require various pre-treatments (mechanical, thermal, or chemical), which usually lead to unfavorable quality changes such as loss of essential nutrients and loss of hardness [9], [10], [11]. An alternative is to use a factor that intensifies the mass-exchange process without significantly interfering with the structure of the fruit.

One novel, advanced technique, which is seen as a potential source for enhancing heat and mass transfer, is ultrasound. These mechanical waves have found a wide range of applications, including the intensification of many diverse operations, for example, osmotic dehydration and drying, as well as vacuum impregnation [12], [13], [14]. The effect of ultrasound depends on many factors: the wave frequency, amplitude and intensity, and the density, viscosity, and temperature of the medium through which they propagate. As most of the ultrasound-assisted processes are carried out in a liquid environment acoustic cavitation also plays the dominant role. The implosion of gas–vapor bubbles leads to a rapid increase in local temperature and pressure (up to hundreds of atmospheres and thousands of Kelvins), resulting in the formation of shock waves, microjets, turbulence, shear forces, etc. [13], [15]. Local pressure fluctuations have a particularly strong influence on the microscopic properties of biological material. The continuous mechanical stress facilitates moisture transport because the molecules are forced to move toward the sample’s surface. This is called the *sponge effect* (a rapid series of alternate compressions and expansions). Furthermore, the formation, growth, and implosion of cavitation bubbles create microchannels, increases the effective porosity of the material being processed, and, thus the volume available during vacuum impregnation. The sudden (local) increase in temperature influences the density and viscosity of solutions and native fluids [16]. In the main, the phenomena induced by ultrasound are intense enough to increase the efficiency of mass and/or heat transfer without causing significant structural changes that could lead to adverse effects in the sample.

Ultrasonically-assisted vacuum impregnation has not yet been investigated. Taking into account the possible benefits, it is reasonable to connect both techniques. Thus, in this study, an attempt was made to combine the impregnation technique with acoustic waves in order to enhance the vacuum impregnation of cranberries, i.e., to fortify them with vitamin C. Many papers can be found concerning the various impregnation methods of soft tissue fruit, but there is very little literature on the use of ultrasound-assisted vacuum impregnation being applied to berry fruits. Cranberries were used during this research as a model, a kind of representative of difficult-to-process fruit.

## 2 Methodology of the study

### 2.1 Material

Cranberries of the variety grown in Poland (*Vaccinium macrocarpon* Aiton cv. Pilgrim) were bought directly from a grower and stored refrigerated at 6 °C, RH 80–85 % for no more than 10 days. Before the impregnation process, the berries were checked for size, ripeness, color, and skin damage. The maturity of the fruit was evaluated on the basis of visual assessment of color and skin condition (bruises, cracks). Only ripe fruit with a diameter of approximately 15–20 mm and undamaged skin were selected for the tests. The weight of a single batch was, on average, 400 g.

### 2.2 Vacuum impregnation

**Fig. 1:**
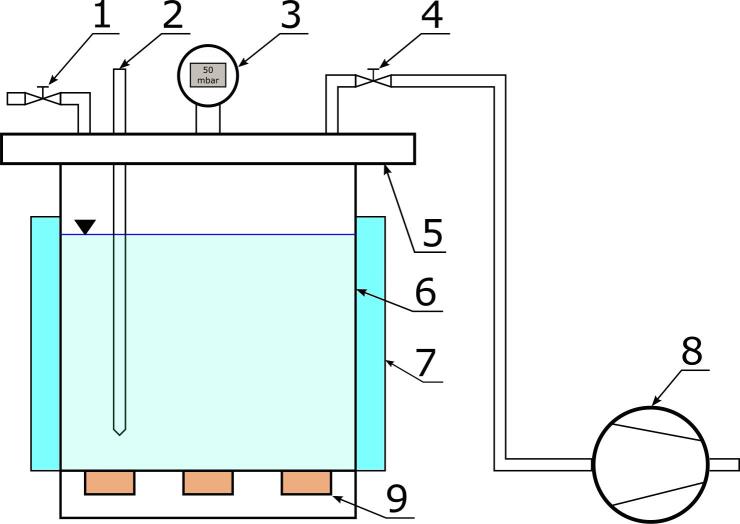
Scheme of impregnation apparatus [17]. 1,4 – valves; 2 – PT100 temperature probe; 3 – vacuum gauge; 5 – lid, 6 – vacuum chamber; 7 – water jacket; 8 – vacuum pump; 9 – ultrasonic transducers.

The vacuum impregnation processes were carried out in an IS-PP apparatus (INTERSONIC Sp. z o.o., Olsztyn, Poland), the diagram of which is presented in Fig. 1.

**Fig. 2:**
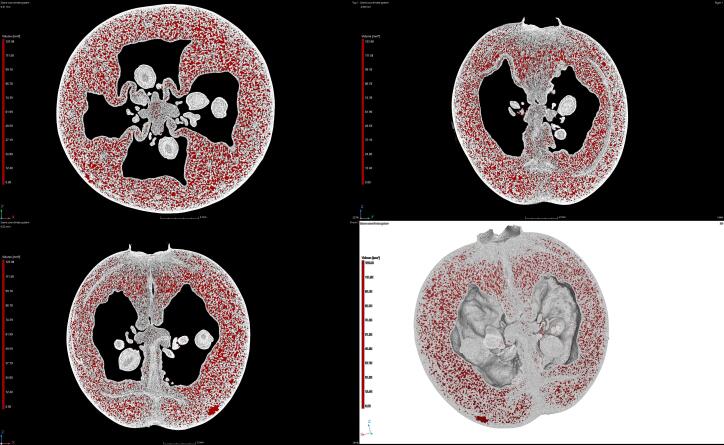
Micro-CT scan of a raw cranberry.

Impregnation was carried out in an aqueous ternary solution composed of analytically pure ascorbic acid (0.5 % w/w), citric acid (0.5 % w/w), and sucrose (9 % w/w) (Chempur, Piekary Śląskie, Poland). The effective concentration of the solution was 10.0 % (w/w): about equal to the cranberries’ soluble solid content (9.8 %) as measured with a PAL-α digital refractometer (ATAGO, Saitama, Japan). The composition of the solution and concentration of the individual components have been selected to stabilize the solution (e.g., prevent the oxidation of ascorbic acid by acidification with citric acid), and to prevent the mass transport from osmosis (ensuring hypertonic conditions by adding sucrose). During each process, 3 kg of solution was used, so the ratio of the weight of raw material to the solution was 1:7.5. The temperature of the solution was measured using a PT100 sensor (Fig. 1, 2) and kept constant at approximately 25 °C using a water jacket (7). In the course of the research, five different impregnation processes were analyzed. Details of the impregnation processes are presented in Table 1. Vacuum impregnation consists of two main periods, which are realized at different pressures (vacuum and atmospheric). Given that the pressure might have influenced the intensity and effectiveness of the ultrasonically induced phenomena, all possible combinations have been analyzed. During the ultrasound-assisted processes (UVI), ultrasonic waves at a frequency of 35 kHz were generated in the solution by piezoelectric transducers (Fig. 1, 9) attached to the bottom of the chamber. The ultrasound’s effective power, measured using the calorimetric method [18], was approximately 240 W.

**Table 1:** Description of the impregnation processes.

Process	Absolute pressure (mbar)		Duration (min.)		Ultrasound power (W)	
	Vacuum	Relaxation	Vacuum	Relaxation	Vacuum	Relaxation
VI	50	atm	30	30	–	–
VI	300	atm	30	30	–	–
UVI-1	300	atm	30	30	240	–
UVI-2	300	atm	30	30	–	240
UVI-3	300	atm	30	30	240	240

VI – vacuum impregnation, UVI – ultrasound-assisted vacuum impregnation, atm – atmospheric pressure.

In UVI-1, ultrasound was applied during the vacuum stage; in UVI-2, during relaxation; and in UVI-3, for the whole process.

The samples were placed in the processing chamber (Fig. 1, 6) into which the impregnating solution was then poured. The lid of the chamber was then closed (5) and a vacuum was generated above the solution using a vacuum pump (8). The pressure in the chamber was measured continuously using a digital pressure gauge (3). When the required value was reached, the valve (4) was closed and the vacuum period started. After the required vacuum time had elapsed, the valve (1) was gradually opened and the chamber was aerated. Once atmospheric pressure was reached, the period of relaxation commenced. After the relaxation time had elapsed, the samples were spread in a single layer on a sieve and allowed to drain for 5 min. The batch was then divided into 2 parts. One part was used to measure the degree of impregnation and color. The other part was placed in a barrier bag, sealed, and frozen at −50 °C. The samples were stored under these conditions until a chemical analysis was carried out on antioxidant capacity, and levels of ascorbic acid, structure compounds, polyphenol, and anthocyanin content.

### 2.3 Ascorbic acid content and impregnation degree

The effectiveness of the vacuum impregnation was assessed on the basis of ascorbic acid content (*AAC*) and impregnation degree (*ID*). Ascorbic acid extraction was carried out with 1 % m-HPO_3_ [19]. The samples were analyzed using an LC Agilent Technologies 1200 Rapid Resolution (Waldbronn, Germany) system, equipped with a Zorbax SB-C18 (4.6 mm × 150 mm; 5 µm) (Agilent Technologies, Santa Clara, CA, USA). Mobile phases used methanol (phase A) and 0.005 mol/L KH_2_PO_4_ solutions (phase B). To detect the ascorbic acid, a gradient of methanol (solvent A) (Sigma Aldrich, Steinheim, Germany) per 0.005 mol/L KH_2_PO_4_ and pH 2.6 (solvent B) (Sigma Aldrich, Tokyo, Japan) was used according to the following program: linear increment starting with 5 % A to 22 % in 6 min., then a return to the initial conditions within the next 9 min., with a flow rate of 0.7 mL/min [20]. The eluate was detected using a UV–vis detector set to 245 nm. The ascorbic acid was identified by comparing its retention time with that of an ascorbic acid standard (Sigma Aldrich, St. Louis, MO, USA).

The impregnation degree (*ID*) was the relative change in the batch mass determined by the relationship:

(1) $$\mathrm{ID}=\frac{m_{i}-m_{0}}{m_{0}}\cdot 100\%$$

where *m*_0_ and *m_i_* are the masses of the material before and after impregnation, respectively.

### 2.4 Color

The color of raw and impregnated cranberries was measured using a Konica Minolta CR400 colorimeter (Tokyo, Japan) and expressed in the CIELAB color space. Before measurement, 10 berries were ground in an IKA A11 Basic (Staufen, Germany) laboratory mill. Next, the obtained paste was placed in a special sample holder and its tristimulus color parameters (L*, a*, b*) were measured five times. This procedure was carried out in 5 replications, so the total number of measurement points was 25. Then, the data were averaged, and, on this basis the total color change (*dE*) was calculated in accordance with the following equation:

(2) $$\mathrm{dE}=\sqrt{{\left(L_{0}^{*}-L_{s}^{*}\right)}^{2}+{\left(a_{0}^{*}-a_{s}^{*}\right)}^{2}+{\left(b_{0}^{*}-b_{s}^{*}\right)}^{2}}$$

where L* indicates lightness, and a* and b* are the chromaticity parameters that indicate color directions: from red to green (a*) and from yellow to blue (b*); index 0 denotes the raw material and *s* denotes the processed sample (impregnated product).

### 2.5 Antioxidant capacity

The radical (ABTS^•+^) 2,2-azinobis-(3-ethylbenzothiazoline-6-sulphonic acid) was produced by reacting 0.007 mol/L ABTS (Sigma Aldrich Chemie Co., St. Louis, MO, USA) water solution with 0.002 mol/L potassium persulphate (Sigma Aldrich Chemie Co., Buchs, Switzerland). The phenolic extract (50 μL) was added to 5 mL of diluted ABTṠ^+^ solution. Then the absorption was measured at 734 nm after 6 min. incubation at 30 °C [21]. The antioxidant activity was expressed as 6-hydroxy-2,5,7,8-tetramethylchroman-2-carboxylic acid (Trolox) (Sigma Aldrich, Steinheim, Germany) in micromoles per 1 g of dry matter (d.m.).

### 2.6 Anthocyanin content

About 10 g of the sample was weighed, and then homogenized using an IKA T-25 homogenizer (Staufen, Germany); it was then extracted with a solvent mixture of methanol, water, and acetic acid (50:48:2 V/V/V). The anthocyanins were separated by means of a LC Agilent Technologies 1200 Rapid Resolution system (Waldbronn, Germany) with a UV–Vis detector (DAD 1260, Waldbronn, Germany). The pigments were separated using a Zorbax SB-C18 (4.6 × 150 mm, 5 µm) (Agilent Technologies, Santa Clara, CA, USA). Solvent A with formic acid (10 %); and solvent B consisting of formic acid (10 %), acetonitrile (30 %), and water (60 %) were used for elution. The gradient increased as follows: 0–8 min., 20 % B; 8–15 min., 40 % B; 15–16 min., 50 % B; 16–20 min., 100 % B. The flow rate was 1 mL/min. The absorbance was monitored at 520 nm and scanned from λ 250 to 600 nm [22]. Anthocyanins were quantified as mg cyanidin-3-O-glucoside equivalent/1 g d.m. (Sigma-Aldrich, St. Louis, MO, USA).

### 2.7 Polyphenol content

Phenols were extracted according to a procedure described by Vallejo et al. [23]. Phenolic compounds were analyzed by means of an LC Agilent Technologies 1200 Rapid Resolution system equipped with a Zorbax, SB-C18 column (4.6 × 150 mm, 5 μm). Acetic acid (60 g/L) in 0.002 mol/L sodium acetate (solvent A) (Piekary Śląskie, Poland) and acetonitrile (solvent B) (Sigma Aldrich Chemie GmbH, Steinheim, Germany) were used for the mobile phase [24]. The run time was 50 min., and the flow rate was 1 mL/min. Separation was conducted using the following gradient program: 0–15 % B for 15 min., 15–30 % B for 25 min., 30–50 % B for 5 min., and 50–100 % B for 5 min. The phenols were quantified at 280 nm, 320 nm, and 360 nm, using the external standard method. Catechin (Sigma-Aldrich, St. Louis, MO, USA), chlorogenic acid, and quercetin (Sigma-Aldrich, Buchs, Switzerland) were used as the standards.

### 2.8 Structure compound content

The content of the acidic dietary fiber (ADF) and the neutral dietary fiber (NDF), both of which consisted of acid detergent lignin (ADL), cellulose, and hemicellulose, was determined using the detergent method described by Dziedzic et al. [25] and van Soest and Wine [26]. Starches were digested using thermostable α-amylase. The cellulose fraction was calculated as the difference between ADF and ADL, whereas the hemicellulose fraction content was calculated as the difference between NDF and ADF. The following chemical reagents were used to estimate the NDF content: neutral disodium versenate dihydrate, disodium tetraborate decahydrate, disodium hydrogen phosphate, ethylene glycol (Poch, Gliwice, Poland), and redistilled water. However, in order to estimate the content of the ADF, 1 N sulfuric acid (Poch, Gliwice, Poland) and *N*-cetyl-*N*,*N*,*N*-trimethylammonium bromide (Merck, Darmstadt, Germany) were used. In addition, sulfuric acid 72 % was used to estimate the ADL content. Analyses were conducted using a Fibertec System M 1020 (Foss, Sweden).

To determine pectin, the freeze-dried material was ground into a powder. A citric acid solution of pH 2.5 was added to the weighed cranberry powder and the sample was heated at 90 °C for 30 min., cooled, and then filtered using Whatman filter paper under vacuum. Ethanol was added to precipitate the pectin. Then, the sample was vacuum filtered and dried at 105 °C, washed with ethanol, and dried again for 24 h at 60 °C [27]. The obtained pectin sample was weighed and the results were expressed as mg of pectin per 100 g d.m.

### 2.9 Microcomputed tomography

A computer tomograph (micro-CT), manufactured by Waygate Technologies (model v|tome|x s240, Wunstorf, Germany), was used to make the images of the fruit. A directional nanofocus X-ray tube allowed high magnifications (up to 200x) to be obtained at a maximum power of 15 W. In order to obtain a good image of the entire cranberry fruit, a magnification was selected that allowed the 3D tomographic image of the whole sample to be completed within 36 min. In order to avoid the fruit drying out, which, otherwise, might have distorted the image, the scanning time was selected experimentally during the preliminary tests. There were 1,200 measurement images taken at a voxel resolution of 23 µm. An X-ray beam with a voltage of 100 kV and a current of 200 µA was used to obtain the images. Porosity was determined based on a reconstruction of the samples (3D model) as the ratio of pores volume to sample volume. The volume of the seed chamber was not taken into account in the calculations.

### 2.10 Statistical analysis

The data presented are mean ± standard deviation. One-way ANOVA and Tukey post-hoc mean comparisons were performed. Additionally, Pearson correlation coefficients (*r*) were calculated. Statistically significant differences at the level of p < 0.05 are marked with different letters. All calculations were performed using Statistica ver. 12 software produced by StatSoft (StatSoft, Tulsa, OK, USA).

## 3 Results and discussion

An X-ray scan of a raw cranberry is presented in Fig. 2.

In Fig. 2, three main parts can be distinguished in the structure of the cranberry fruit: the skin, the flesh, and the seed chamber. The skin is characterized by a very compact structure and low porosity. This part absorbs the most radiation, as evidenced by the uniform white color in the photo. Under the skin there is a tissue that is less dense and much more porous. The last part, the seed chamber, is filled with air and takes up a significant part of the fruit. The porosity of the whole fruit, based on calculations from the 3D model, is about 23.58 ± 1.17 % (Fig. 2, red area). It is difficult to compare this value with the literature data, because in most papers, only the apparent porosity (which takes into account the volume of the seed chamber) is calculated. Zielińska et al. [28] and Staniszewska et al. [29] determined the apparent density of cranberries gravimetrically at 52 ± 1 %. Similar results were observed by Vagenas et al. [30] for seedless sultana grapes: a porosity of 46 %. On the other hand, Nowak et al. [31] determined the porosity of raw blueberries based on the true density of the fruit, and stated that it was equal to only 6.2 ± 1.7 %. This confirms that the volume of the seed chamber is important and needs to be taken into account during porosity calculations.

**Fig. 3:**
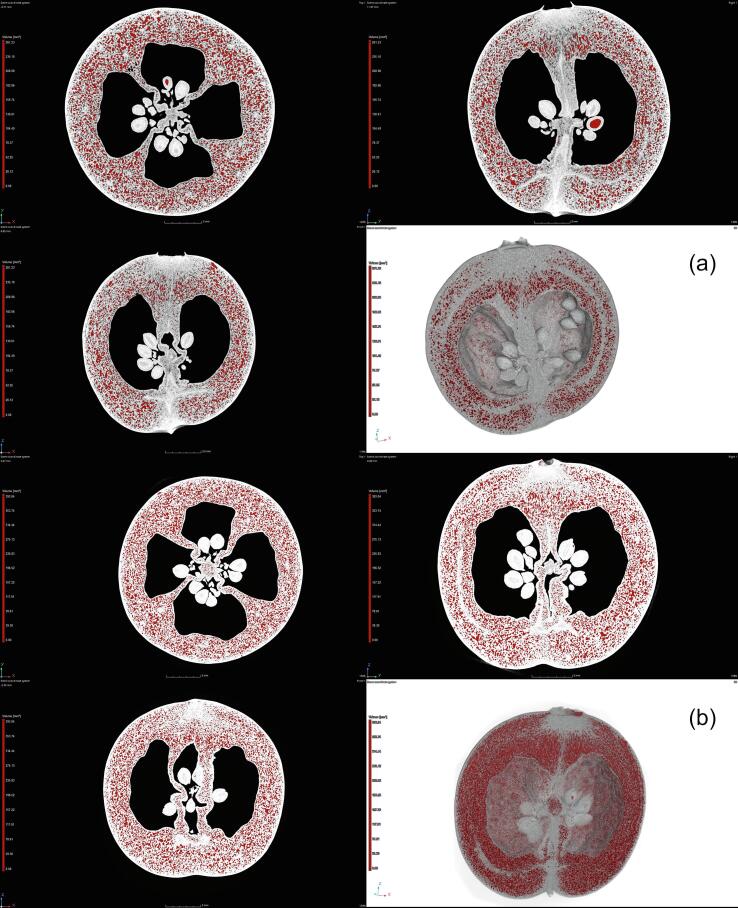
Micro-CT scans of cranberries subjected to vacuum impregnation at 50 (a) and 300 (b) mbar.

Fig. 3 shows scans of cranberry fruits subjected to vacuum impregnation for the same length of time but under different pressures. The images differ visibly. Compared to the raw fruit (Fig. 2), the samples impregnated at 50 mbar are characterized by lower porosity, 15.16 ± 1.56 % on average (Fig. 3a, red area), which may imply that the impregnating solution filled a greater volume than was previously occupied by the native fluids. The samples processed at 300 mbar have a porosity comparable to the raw material, that is 22.96 ± 1.92 % on average (Fig. 3b, red area). So, it may be assumed that the impregnating solution has just replaced the native fluids removed during the vacuum stage.

**Fig. 4:**
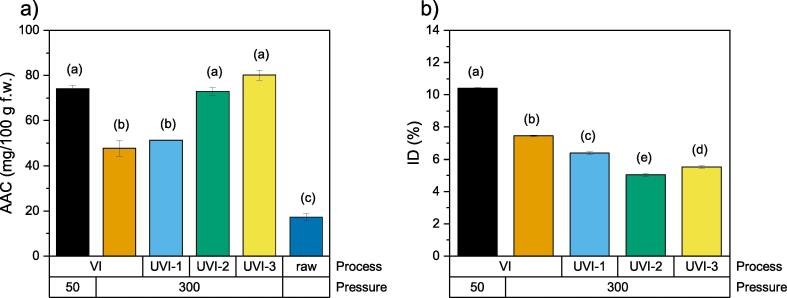
Ascorbic acid content AAC (a) and impregnation degree ID (b). Values are mean ± standard deviation; the different letters above the bars indicate a significant difference at p < 0.05 for the ANOVA and Tukey post-hoc test mean comparisons.

The analysis of ascorbic acid content (*AAC*) confirms the assumptions previously made (Fig. 4a). In the process carried out at 50 mbar, the *AAC* was 74 ± 5 mg/100 g, while for samples processed at 300 mbar, it decreased to 48 ± 11 mg/100 g. Taking into account that the ascorbic acid content in raw cranberries is 17 ± 5 mg/100 g, vacuum impregnation caused a significant increase in *AAC* content: 362 % and 193 % at 50 and 300 mbar, respectively. These results clearly show that whole, un-pretreated cranberry fruit may be successfully enriched with ascorbic acid through vacuum impregnation. The effectiveness of the process depends strictly on the pressure applied. Using higher vacuum delivers greater *AAC* increases. However, operating at low pressure is not favorable for technical and economic reasons, and may lead to negative quality changes in the fruit. As reported by Fito et al. [7], higher vacuum pressure values may cause irreversible tissue deformation or tissue relaxation, and may even result in reduction of free space for the impregnating solution. Therefore, an attempt was made to intensify the impregnation process – enhance the mass transfer – with less vacuum (i.e., higher pressure; 300 mbar) through the use of ultrasound.

In Fig. 4, diagrams showing ascorbic acid content (*AAC*) and degree of impregnation (*ID*) for both raw and impregnated samples are presented.

The results clearly showed that the effectiveness of ultrasound depends on the period of application. The highest *AAC* was noted for UVI-3/300, with a value similar to that observed in the VI process at lower pressure (Fig. 4a, VI/50), which surely results from the long-term action of the mechanical waves. The lowest *AAC* was observed for UVI-1/300 – a value similar to that observed for VI/300 (Fig. 4a). These results may indicate that two different types of cavitation occurred during ultrasound-assisted vacuum impregnation. Acoustic cavitation may proceed without the implosion of gas–vapor bubbles, this is termed *stable cavitation*, while cavitation with implosion is *unstable cavitation* [15]. The dominant type of cavitation depends strictly on the properties of the medium through which the ultrasound propagates, as well as the characteristics of the waves themselves [32]. Unstable cavitation is dominant when the pressure acting on the gas–vapor bubbles exceeds the value of the surface tension. This means that the cavitation threshold has then been exceeded. When ultrasound was applied in the vacuum stage at 300 mbar (UVI-1), the cavitation threshold was probably not exceeded due to the low pressure in the chamber. Stable cavitation was dominant, and caused a small intensification of the mass transfer. Its primary role was probably “ordering” the outflow of the native fluids from the pore spaces. The application of ultrasound at atmospheric pressure (during relaxation - UVI-2/300) caused a significant increase in *AAC* due to unstable cavitation’s dominant role. Thus, a greater intensification of mass transfer was caused by the numerous phenomena that occurred during the implosion of gas–vapor bubbles. The small difference between UVI-2 and UVI-3 (at 300 mbar) may confirm these assumptions. The longer ultrasound time did not cause any significant change in *AAC* due to the dominant role of stable cavitation in the vacuum stage.

The results for *ID* (Fig. 4b) confirmed that each of the impregnation processes led to mass gain in the fruit; but, overall, the *ID* can be considered to be rather small. The highest degree of impregnation was found for process VI at 50 mbar (10.0 %), while for the other ultrasound assisted processes, a downward trend was observed (7.5–5.0 %). A smaller *ID* in the ultrasonically-treated samples may have resulted from the berries’ surfaces being cleaned (e.g., from wax), meaning a loss in mass. Irazoqui et al. [33] observed such an effect on lettuce surfaces. On the other hand, Vasile et al. [34] noted that a significant increase in water content was lacking (mass of samples) in apple tissue during ultrasound-assisted impregnation using a hypotonic solution containing vitamin B12. Substantial simultaneous impregnation of the tissue with vitamin B12 was found.

As there is no strict connection between *AAC* and *ID*, some additional phenomena must be involved during ultrasound-assisted impregnation. One of the possibilities is osmosis. Long relaxation period can cause a local change in osmotic pressure. It could result in dehydration of the tissue (main flux) and an increase in the soluble solids in the material being processed (counter concurrent flux). Osmosis phenomena will be particularly promoted during ultrasound-assisted impregnation because of stirring, microstreaming, pressure, and temperature alteration drop in density and viscosity of the fluids and reduction of the boundary layer [35].

**Fig. 5:**
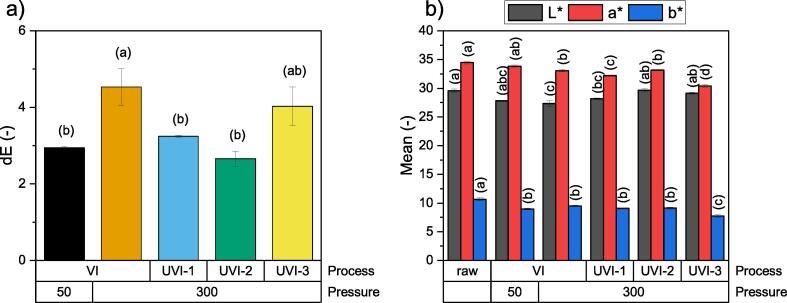
Relative color change, dE (a) and color components L*, a* and b* (b). Values are mean ± standard deviation; the different letters above the bars indicate a significant difference at p < 0.05 for the ANOVA and Tukey post-hoc test mean comparisons.

The intensification of unit operations often takes place at the expense of other parameters, causing, for example, a deterioration in the quality of the products. One of the quality features that was assessed first was color. In Fig. 5, the relative color change, *dE*, and color components L*, a* and b* are presented.

The *dE* value indicates the vector length between two points within the three-dimensional CIELAB color space. The greater the value, the longer the vector, i.e., the greater the difference between the color of the sample (impregnated product) and the standard (raw material). It is generally assumed that a normal observer should be able to perceive a difference in color when *dE* is equal to unity. However, due to the many factors that affect perception of color, a *dE* value equal to 4 is more often defined as the border at which an observer notices a clear difference in color [36]. Only in two samples, *dE* exceeded 4, namely VI/300 and UVI-3/300 (Fig. 5a). In the case of the VI/300 sample, the color difference is caused by a decrease in all color coordinates (Fig. 5b). In turn, in the UVI-3 sample, the color change results mainly from the decrease in chromatic parameter values, a* and b*. This may indicate that the use of ultrasound has a different influence on the color parameters than vacuum impregnation. All the samples treated with ultrasound were characterized by less color difference. However, more research is needed to confirm this effect.

The next quality parameter to be assessed was the anthocyanin and polyphenol content. Some studies have indicated that there is the possibility that anthocyanins are degraded by reactive oxygen species generated during ultrasound treatment [37]. However, no influence on the total anthocyanin content in cranberry fruit was observed from the application of vacuum pressure or ultrasound (Table 2). In the conducted study, the total anthocyanin content in the impregnated fruit was similar to that in the raw material; thus, no sonolysis effect was observed. Only cyanidin-3-O-glucoside was found to be of higher content in the UVI-3/300 sample. This may indicate an improvement in the extractability of this compound from the raw material caused by the use of ultrasound. Chen et al. [38] indicated a loosening of the berries’ structure and an increase in the number of ruptured cells during ultrasonic treatment. This was due to acoustic cavitation and microjets, which can damage cellular material. In the previous studies [39] conducted on cranberry halves, a decrease in anthocyanin content was observed in the impregnated samples. Comparing the whole fruit (current study) with the halves (previous study), the *ID* amounted to 5–10 % and 36–52 %, respectively. Thus, in the case of whole fruit, especially for the UVI-2 and UVI-3 (at 300 mbar) samples, it is possible to obtain a satisfactory level of vitamin C fortification while avoiding negative impacts on the pigment content of the raw material. The results confirmed the observation made by instrumental color measurement: the differences in color between individual samples were negligible (see Fig. 5a).

**Table 2:** Anthocyanin content.

Compound (mg/g d.m.)	Process/pressure (mbar)					RAW
	VI/50	VI/300	UVI-1/300	UVI-2/300	UVI-3/300	
cyanidin-3-O-galactoside	1.92 ± 0.16 a	1.92 ± 0.04 a	1.84 ± 0.18 a	2.02 ± 0.02 a	2.12 ± 0.04 a	1.92 ± 0.18 a
cyanidin-3-O-glucoside	5.9 ± 6·10^-3^b	5.4 ± 6·10^-3^ ab	4.6 ± 4·10^-3^ a	5.0 ± 4·10^-3^ ab	9.6 ± 6·10^-3^c	4.8 ± 4·10^-3^ ab
cyanidin-3-O-arabinoside	1.68 ± 0.16 a	1.62 ± 0.02 a	1.60 ± 0.18 a	1.64 ± 0.02 a	1.74 ± 0.10 a	1.50 ± 0.12 a
peonidin-3-O-galactoside	2.72 ± 0.20 a	2.40 ± 0.04 a	2.48 ± 0.24 a	2.68 ± 0.02 a	2.84 ± 0.04 a	2.62 ± 0.28 a
peonidin-3-O-glucoside	0.26 ± 0.02 a	0.24 ± 0.02 a	0.24 ± 0.02 a	0.24 ± 0.02 a	0.34 ± 0.02 a	0.24 ± 0.02 a
peonidin-3-O-arabinoside	1.16 ± 0.14b	1.38 ± 0.04 ab	1.42 ± 0.14 ab	1.40 ± 0.02b	1.56 ± 0.04b	1.24 ± 0.10 a
Total	8.2 ± 0.3 a	7.6 ± 0.2 a	7.6 ± 0.8 a	8.0 ± 0.1 a	8.7 ± 0.3 a	7.6 ± 0.4 a

Values are mean ± standard deviation; the different letters in each column indicate a significant difference at p < 0.05 for the ANOVA and Tukey post-hoc test mean comparison.

The phenolic content in both the raw material and the impregnated samples was in the range of 13.6–20.7 mg/g d.m. (Table 3). These results remain in accordance with data provided by other researchers [40], [41], [42]. The vacuum impregnation process had no significant effect on the total phenolic content in the samples, regardless of the pressure applied. However, the application of ultrasound during vacuum impregnation resulted in a significantly higher phenolic content. Changes in the microstructure induced by ultrasound allowed for enhanced extraction. The effects of ultrasound on improving the extractability of phenolic compounds from plant tissue has been described in numerous studies [43], [44], [45]. Moreover, the thick skin of the cranberry prevents leakage after the vacuum impregnation process, thus retaining compounds in the tissue. The highest phenolic compound content was recorded when using ultrasound during the entire process (UVI-3/300). Samples subjected to ultrasound treatment only during the vacuum stage (UVI-1/300) or the relaxation stage (UVI-2/300) had a lower polyphenol content. Similarly, in studies by Yılmaz and Bilek [14], bioactive compounds in apple discs were lower when ultrasound only operated during the vacuum or relaxation period of process VI as opposed to the entire process. In studies by Khan et al. [44], no degradation of phenolic compounds was reported during their extraction from orange peel using ultrasound treatment. However, some authors have indicated the possible degradation of phenolic compounds after using sonication, due to the formation of Reactive Oxygen Species (ROS) during cavitation. This was noted, for example, in apples [46], parsley leaves [47], and melon juice [48] after treatment was applied, for example, contact sonication.

**Table 3:** Polyphenol content.

Compound (mg/ g d.m.)	Process/Pressure (mbar)					RAW
	VI/50	VI/300	UVI-1/300	UVI-2/300	UVI-3/300	
Epicatechin	1.76 ± 0.09b	1.34 ± 0.08 a	2.47 ± 0.07c	2.50 ± 0.07c	2.86 ± 0.13 d	1.14 ± 0.08 a
Procyanidins	1.5 ± 0.4 a	1.9 ± 0.1 ab	2.0 ± 0.3 ab	2.0 ± 0.1 ab	2.6 ± 0.5b	1.6 ± 0.2 a
Caffeic acid	0.27 ± 0.02 a	0.35 ± 0.06 bc	0.46 ± 0.06 bc	0.33 ± 0.08 ab	0.55 ± 0.07	0.27 ± 0.03 a
Chlorogenic acid	1.14 ± 0.09 a	1.28 ± 0.14 a	1.32 ± 0.14 a	1.34 ± 0.10 a	1.36 ± 0.09 a	1.20 ± 0.13 a
P-Cumaric acid	2.19 ± 0.06 ab	2.37 ± 0.05b	2.35 ± 0.08b	2.30 ± 0.06 ab	2.66 ± 0.06c	2.16 ± 0.07 a
Ferulic acid	0.25 ± 0.02b	0.20 ± 0.02 ab	0.36 ± 0.01c	0.37 ± 0.01c	0.41 ± 0.04c	0.15 ± 0.01 a
Myricetin	0.65 ± 0.07 a	1.15 ± 0.08 ab	0.66 ± 0.01 a	0.67 ± 0.05 a	0.72 ± 0.06 a	1.00 ± 0.11 ab
Quercetin	6.5 ± 0.2b	5.2 ± 0.1 a	7.1 ± 0.2c	7.4 ± 0.2 d	9.5 ± 0.2 d	6.7 ± 0.2b
Total	13.6 ± 0.9 a	13.8 ± 1.2 a	16.7 ± 0.9 bc	16.9 ± 0.7c	20.7 ± 1.2 d	14.2 ± 0.8 ab

Values are mean ± standard deviation; the different letters in each column indicate a significant difference at p < 0.05 for the ANOVA and Tukey post-hoc test mean comparison.

Antioxidant capacity was the next parameter assessed in this study. Results showed that the parameter’s value in the raw material was significantly lower than the other samples (Table 4). In the vacuum impregnated samples, the antioxidant activity was 481 and 462 µmol/g d.m., for 50 mbar and 300 mbar, respectively. On the other hand, the use of ultrasound in the UVI process resulted in values ranging from 435 to 528 µmol/g d.m.

**Table 4:** Antioxidant activity.

Process/Pressure (mbar)	Antioxidant capacity (µmol/g d.m.)
VI/50	481 ± 20 bc
VI/300	462 ± 19 bc
UVI-1/300	435 ± 8b
UVI-2/300	460 ± 24b
UVI-3/300	528 ± 28c
RAW	313 ± 36 a

Values are mean ± standard deviation; the different letters in the column indicate a significant difference at p < 0.05 for the ANOVA and Tukey post-hoc test mean comparison.

The highest antioxidant capacity values were characteristic of the samples subjected to ultrasound treatment during the entire process (UVI-3/300), and were significantly higher than the values obtained when using ultrasound in the vacuum (UVI-1/300) or relaxation stages (UVI-2/300). Yılmaz and Bilek [14] reported similar results for the vacuum impregnation of apple tissue. The use of ultrasound during the entire vacuum impregnation process resulted in a higher antioxidant capacity in apples than when it was applied only during the vacuum or relaxation periods.

The antioxidant capacity of cranberry sample UVI-3/300 did not differ significantly from the values obtained for impregnated samples (for both pressure values) without ultrasound treatment. This distribution of values is consistent with the ascorbic acid content (see Fig. 4a). A statistically significant, strong correlation (*r* = 0.926) was found between the antioxidant capacity and the content of ascorbic acid in the samples. However, a weaker correlation was found between the antioxidant capacity of the samples and both the phenolic compound content (*r* = 0.51) and the anthocyanin content (*r* = 0.716). In a study by Nowacka et al. [49], it was noted that the ultrasonic treatment of whole cranberries retained the antioxidant activity at a similar level to the untreated samples. This is consistent with the results obtained in this study, taking into account the increase in antioxidant activity due to the impregnation of the tissue with ascorbic acid and the lack of correlation in regard to the polyphenol and anthocyanin content.

The results for the *AAC* and *ID* clearly indicated that ultrasound-induced structural changes took place; however, no determination was able to be made based on the microtomography images. For this reason, the content of structure-forming compounds in samples impregnated without ultrasound (VI/50 and VI/300) and after the most intensive ultrasound action (UVI-3/300) was determined (Table 5). No significant changes in the content levels of most of the structure-forming compounds were found, either at lower pressure (50 mbar) or after the application of ultrasound. The only decrease was for hemicellulose in the ultrasound-assisted process (UVI-3/300). Renouard et al. [50] found that ultrasound has the ability to degrade hemicellulose. The extraction of hemicellulose from plant materials can also be improved by applying ultrasound [51]. Bussemaker and Zhang [52] indicated that cavitation causes the formation of free radicals which are capable of degrading carbohydrates through dissociating their molecules. Moreover, it is possible that high speed microjets produce a mechanical effect with the ability to break cell walls. The low levels of hemicellulose in the UVI-3/300 samples could be related to tissue structure damage and the outflow of hemicelluloses or their degradation. This effect could also contribute to the highest levels of the phenolic and anthocyanin compounds’ content in the UVI-3/300 samples.

**Table 5:** Structure-forming compounds.

Compound (g/ 100 g d.m.)	Process/Pressure (mbar)			RAW
	VI/50	VI/300	UVI-3/300	
ADF	15.9 ± 0.5 a	16.0 ± 0.6 a	16.5 ± 0.4 a	16.6 ± 0.5 a
NDF	20.3 ± 0.6 ab	21.4 ± 0.7 ab	19.9 ± 0.8 a	22.4 ± 1.4b
ADL	6.9 ± 0.4 a	6.9 ± 0.8 a	7.8 ± 0.2 a	8.0 ± 0.6 a
cellulose	9.0 ± 0.6 a	9.1 ± 0.4 a	8.7 ± 0.6 a	8.6 ± 0.7 a
hemicellulose fraction	4.4 ± 0.4 ab	5.4 ± 0.8 ab	3.4 ± 0.4 a	5.8 ± 1.0b
pectin fraction	3.3 ± 0.2 a	3.3 ± 0.2 a	3.6 ± 0.4 a	3.2 ± 0.4 a

ADF – acidic dietary fiber, NDF – neutral dietary fiber, ADL – acid detergent lignin.

Values are mean ± standard deviation; the different letters in each column indicate a significant difference at p < 0.05 for the ANOVA and Tukey post-hoc test mean comparison.

## 4 Conclusions

In this study, the quality and microstructural properties of berry fruits impregnated with different methods were investigated. Cranberry and an aqueous solution containing ascorbic, citric acid, and sucrose have been tested as a model system for studying the migration of various substances to or from the inside tissue structure without and under sonication. The use of microtomography allowed the true porosity of cranberry fruit to be determined, which, with the seed chamber, amounts to approximately 24 %. Thus, this space provides the opportunity to modify the composition of this raw material using vacuum impregnation. The characteristics that make the impregnation of cranberry tissue difficult is its thick skin and the layer of wax on the fruits’ surface. The conducted research shows it is possible to effectively impregnate the tissue with ascorbic acid using vacuum impregnation at a pressure of 50 mbar, or using ultrasound during either the relaxation stage or the entire process at 300 mbar. It can be assumed that the ultrasound-assisted impregnation process contributed to the enrichment of the tissue with ascorbic acid by a mechanically induced pressure change, but also as a result of supporting diffusion during the relaxation stage. It was also found that the application of ultrasound during the vacuum stage is more likely to cause stable cavitation (without implosion). But, due to the cyclic pulsations in the medium, it organized the flow and slightly reduced the boundary layer. In turn, ultrasound under atmospheric pressure caused unstable cavitation (with implosion), which significantly affected the mass transfer during vacuum impregnation. Ultrasound-assisted impregnation (UVI) as a combined technique shows promising results taking into account the effects strengthening the fortification of difficult-to-process fruit with food additives.

## CRediT authorship contribution statement

**Dominik Mierzwa:** Conceptualization, Formal analysis, Investigation, Methodology, Project administration, Visualization, Writing – original draft, Writing – review & editing. **Justyna Szadzińska:** Investigation, Writing – original draft, Writing – review & editing. **Bartosz Gapiński:** Formal analysis, Investigation, Methodology, Visualization. **Elżbieta Radziejewska-Kubzdela:** Conceptualization, Formal analysis, Investigation, Methodology, Project administration, Writing – original draft, Writing – review & editing. **Róża Biegańska-Marecik:** Investigation, Methodology, Writing – review & editing.

## Declaration of Competing Interest

The authors declare that they have no known competing financial interests or personal relationships that could have appeared to influence the work reported in this paper.

## Data Availability

Data will be made available on request.
